# A Rare Presentation of Superior Mesenteric Artery Syndrome as Acute Abdomen

**DOI:** 10.7759/cureus.31484

**Published:** 2022-11-14

**Authors:** Husain M Alaradi, Hasan R Juma, Zainab M Abdulnabi, Mohamed Isa

**Affiliations:** 1 Surgery, Salmaniya Medical Complex, Manama, BHR; 2 General Surgery, Salmaniya Medical Complex, Manama, BHR

**Keywords:** acute abdomen, superior mesenteric artery, intestinal obstruction, vomiting, abdomen pain, weight-loss

## Abstract

Small bowel obstruction has many etiologies, but superior mesenteric artery syndrome (SMAS) is among the rarest causes. It happens when the third part of the duodenum is compressed between the superior mesenteric artery and the abdominal aorta, preventing gastric content from passing through the small intestine. SMAS is a diagnosis of exclusion because it is atypical and needs a high index of suspicion. There is frequently a delay in diagnosis, leading to morbidity and mortality. We present a case of a young female who presented with symptoms of episodic abdominal pain and obstruction. A computed tomography scan revealed SMAS. She was admitted and treated conservatively with total parenteral nutrition for one week and intravenous fluids, and eventually, her bowel opened, and the condition resolved.

## Introduction

Superior mesenteric artery syndrome (SMAS) is a rare but life-threatening gastrointestinal disease. It develops when the typical 45° angle between the superior mesenteric artery (SMA) and the abdominal aorta (AA) is reduced from 6° to 25° [1]. The incidence of SMAS is estimated at 0.1% to 0.3%, with the usual age of presentation being 10 to 39 years, with a male-female ratio of 3:2, or predominance in female patients [2-4]. SMAS is considered a diagnosis of exclusion, and common symptoms include vomiting, nausea, and epigastric abdominal pain. Accurately diagnosing SMAS can be challenging, given that other disorders can produce the same symptoms. There are only a small number of cases reported in our literature search so far in which it was presented as acute abdomen with intestinal obstruction. We diagnosed her with CT abdomen and pelvis with IV contrast and then treated her conservatively over one week with good outcomes.

## Case presentation

A 31-year-old woman with a significant past medical history of multiple sclerosis was presented to a private hospital with a four days history of severe upper abdominal pain and distention. She reported the pain was crampy with intermittent pain not radiating, there were no relieving factors, and it increased with meals and when lying down. It was associated with several episodes of vomiting and nausea. She did not pass stool or gases for three days and denied a history of weight loss or constitutional symptoms.

Her medication included Fingolimod hydrochloride 0.5 mg OD for multiple sclerosis.

She appeared conscious, alert, and in distress due to pain but had stable vital signs. Her height was 155 cm, and she weighed 45 kilograms with a BMI of 18.7. An abdominal examination showed severe distention with diffuse tenderness and guarding.

Her blood results showed serum sodium of 140 mmol/L, magnesium of 0.91 mmol/L, potassium of 4.1 mmol/L, albumin 4.1 g/dl, creatinine 0.61 mg/dl, amylase 54 (U/L), white cell count of 8.3 109/L, and hemoglobin 10 g/dl.

**Figure 1:**
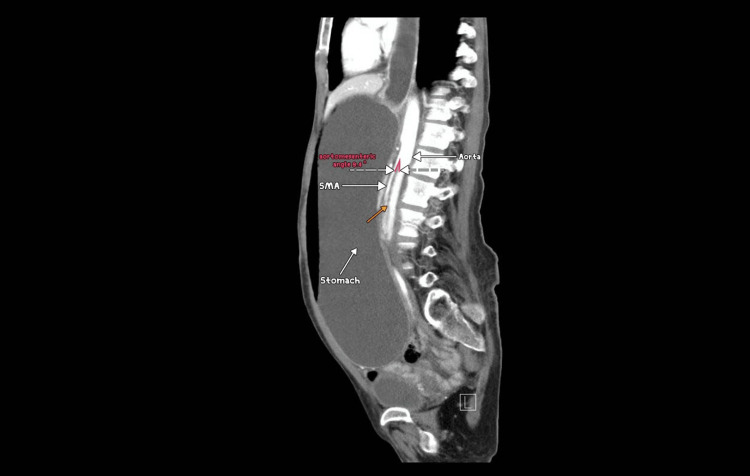
CT showing duodenum obstruction (orange arrow) between the aorta and SMA. CT: Computed tomography; SMA: Superior mesenteric artery

**Figure 2:**
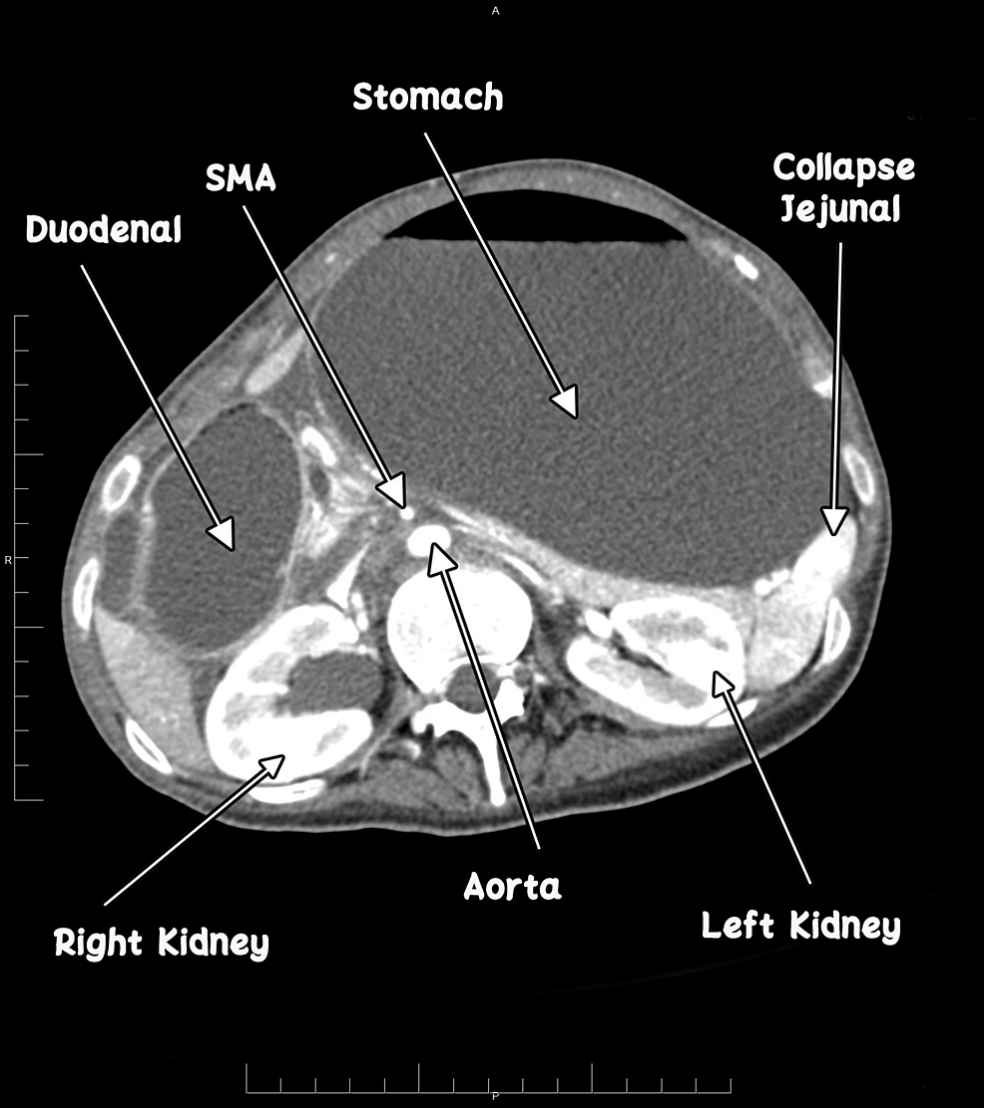
Collapsed jejunal loops distal to the obstructing point.

**Figure 3:**
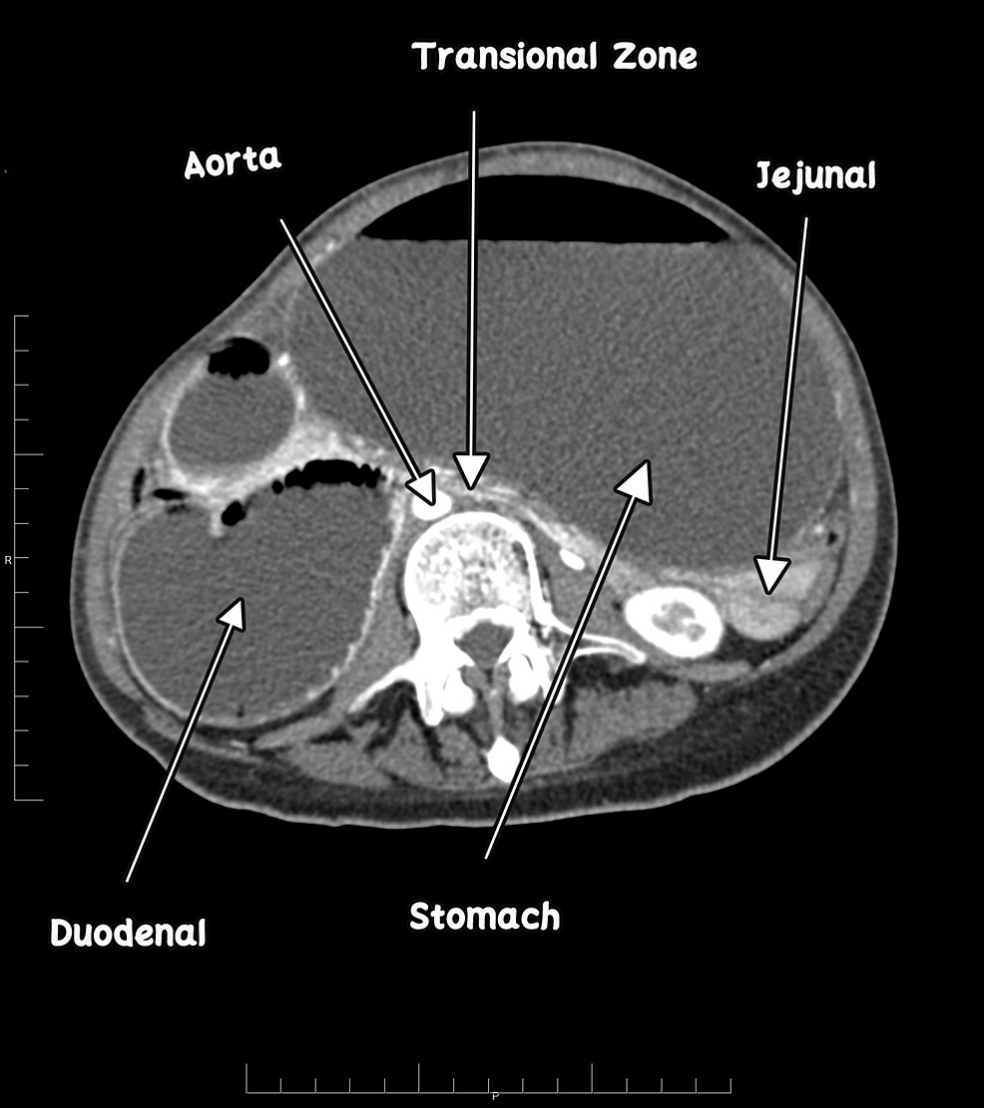
Transitional zone between dilated obstructed duodenum and collapsed jejunal loops.

**Figure 4:**
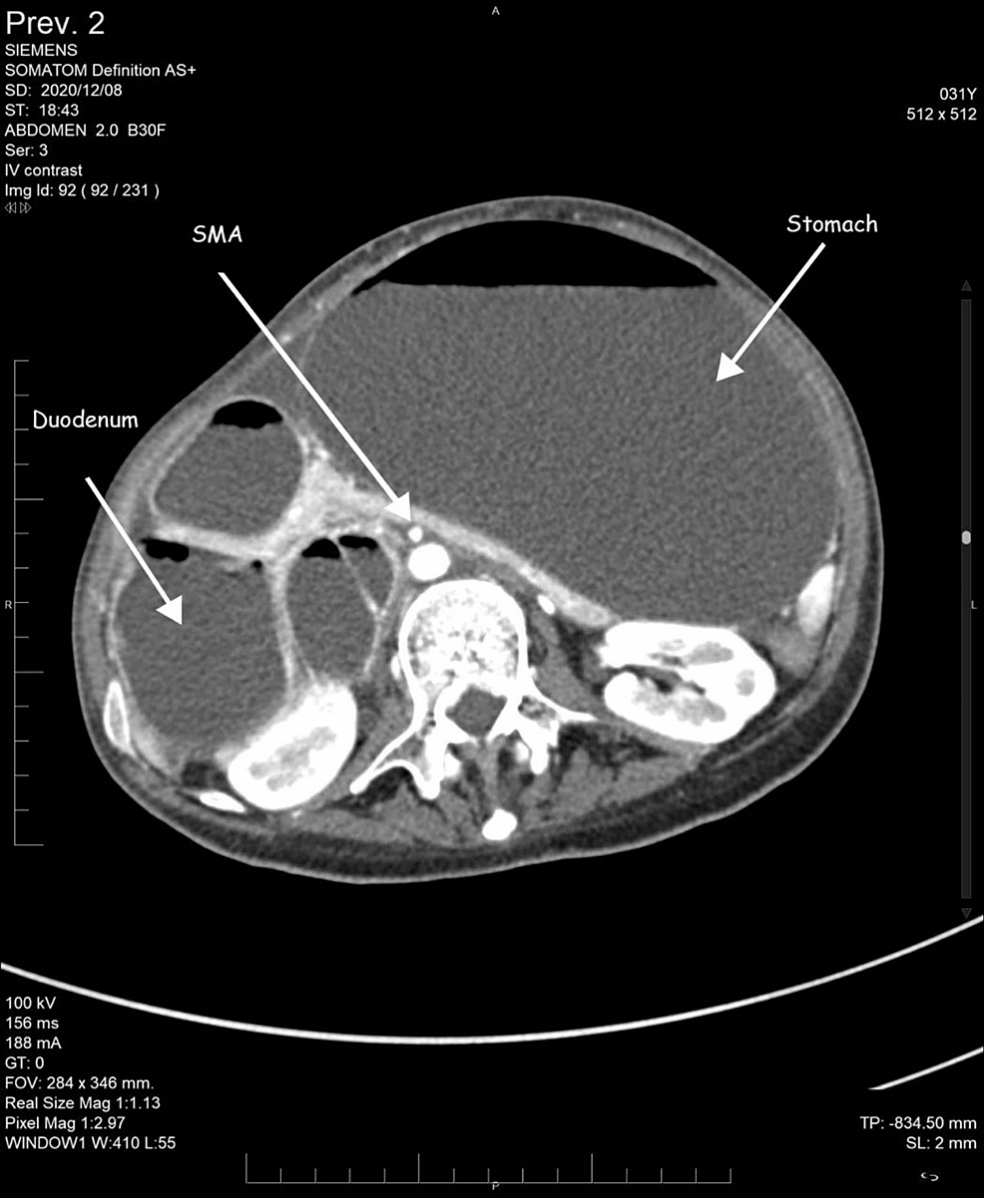
Axial contrast-enhanced CT of the abdomen showing a grossly dilated stomach and proximal duodenum. CT: Computed tomography; SMA: Superior mesenteric artery

**Figure 5:**
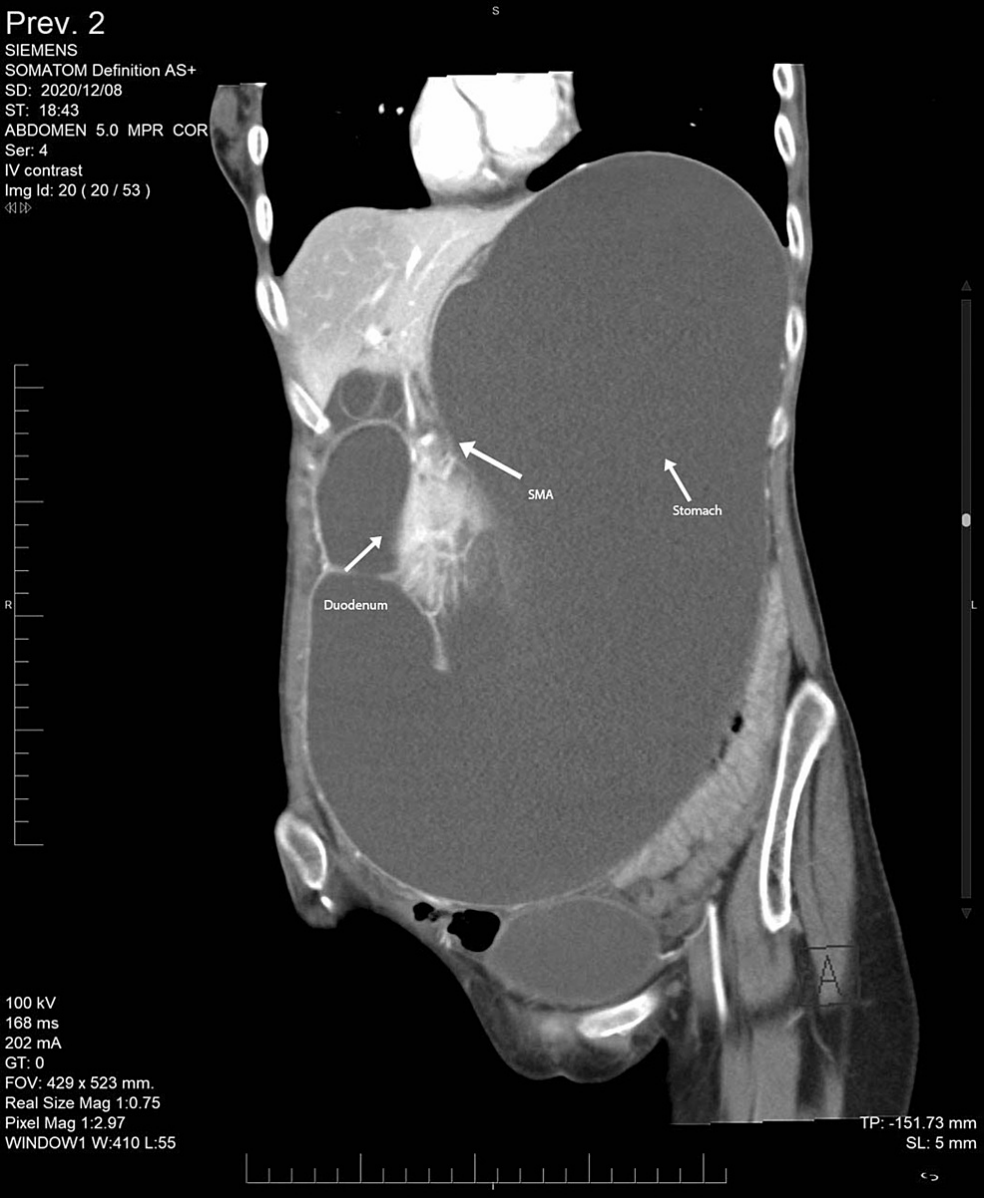
CT showing grossly distended stomach and duodenum. CT: Computed tomography; SMA: Superior mesenteric artery

A computed tomography (CT) scan of her abdomen and pelvis was obtained with IV/oral contrast (Figures 1-5).

It showed gross distention of the stomach with a measurement of 28.37 cm × 11.34 cm × 14.87 cm and the first and second parts of the duodenum with an abrupt cut-off sign in the third part of the duodenum. Sagittal sections revealed that both parameters are reduced angles of 9.4° and 5.29 mm between SMA and the abdominal aorta, compressing the third part of the duodenum. Axial contrast showed collapsed jejunal loops distal to the obstructing point with a transitional zone between dilated obstructed duodenum and collapsed jejunal loops. No free fluid or free air was noted.

The patient was admitted and resuscitated intravenously with electrolyte and analgesic therapy. The nasogastric (NG) tube was positioned with 2.6 liters of drained bilious liquid, and the upper endoscopy showed a normal esophagus and stomach. The NG tube has been free-flowing with five hourly vacuum cleaners, which helped with her symptomatic control. We were confident that she had SMAS, and we decided to treat it conservatively with total parenteral nutrition (TPN). 

Following a regular dietary review, enteral feeding began after one week of TPN, and the rate slowly raised until the target rate was reached. The enteral feeding was increased with a regular dietician review, and five cans of Ensure Plus per day were given, which contained 1500 kcal and 62.5 grams of protein. She received a gastrografin challenge that revealed no further bowel obstruction. Finally, it was decided that once the patient could tolerate more oral foods, she should maintain a continuous oral diet, and her health would continue to improve. Following her discharge, she was seen by her regular neurologist physician. In her three months, she attained her usual weight of 49 kg, tolerated a regular diet, and relieved the symptoms.

## Discussion

SMAS (also called Wilkie's syndrome) is a rare etiology of third-party duodenal compression between SMA and AA. Typically, the AA and SMA are at a 38° to 56° angle and a 10 to 20 mm distance. SMAS is diagnosed when the angle is reduced to less than 25 degrees. It is also supported by the finding of a dilated stomach and first and second portions of the duodenum, and a decompressed bowel distally. The condition usually results from the loss of fat pads between SMA and AA, which happens with rapid weight loss. It is usually seen with catabolic conditions like tuberculosis, cardiac cachexia, anorexia nervosa, malignancies after bariatric surgery, drug abuse, post-esophagectomy, malabsorption states, etc. [5-6].

In cases of rapid weight loss involving intra-abdominal adipose tissue, the loss of such intra-abdominal fat narrows the AA to SMA angle, causing a mechanical obstruction. If the angle is reduced from 6° to 25°, SMAS develops [1]. Acute intestinal obstruction and chronic unspecific abdominal symptoms (e.g., postprandial fullness, early satiety, recurrent episodes of abdominal pain, and cramping) are among the symptoms [7]. Patients might report concerns about abdominal pain aggravated by lying supine and relieved by left lateral decubitus or even in the knee-chest position, which increases the aortomesenteric angle and potentially reduces the small bowel mesentery tension [8].

The diagnosis is usually delayed because of the presentation's non-specific nature and the condition's rarity. An elevated level of clinical suspicion and a detailed evaluation of CT images needs to be performed. In equivocal cases, the point of obstruction can be elucidated by doing upper GI contrast studies to demonstrate obstruction or slow passage of contrast in the third part of the duodenum [9].

Conservative medical management is the initial management for SMAS and aims to resolve the underlying cause.

The initial treatment should focus on resuscitation, electrolyte replacement, and TPN with a slow increase of cal/kg to avoid refereeing syndrome. The aim is to gain some weight to widen the SMA-AA angle. Nutritional support is especially required in the initial stages until the patients can tolerate more oral food to increase their oral intake. It is usually provided by placing a nasojejunal feeding tube distal to the site of obstruction [10]. Total parenteral nutrition can be used in patients who cannot tolerate enteral foods or when they seek higher caloric supplementation. If conservative treatment fails, surgical treatment is possible. The status of routine dietary reviews should be reassessed before surgery to guarantee a positive result.

There are three types of surgical procedures used to treat SMA syndrome. The Strong procedure is the least invasive surgery because it does not need bowel anastomosis. The procedure involves dividing the ligament of Treitz and mobilizes the duodenum, allowing the duodenum to fall away from the aorta [11]. However, Strong's procedure fell out of favor because of its high failure rate. The high failure rate is presumably due to short branches of the inferior pancreaticoduodenal artery not permitting the duodenum to fall inferiorly [12]. The most commonly performed procedure to treat SMAS is Duodenojejunostomy, and it is also the most successful surgical procedure. Gastrojejunostomy may also be performed if gastric distension is still present.

A literature review compiles details regarding symptoms, management, and at the time of follow-up to resolve the symptoms. A total of 13 cases were identified for 10 years, from January 2010 to November 2020 (Table 1).

**Table 1:** Literature review compiling details regarding symptoms, treatment and duration follow-up of SMA syndrome. SMA: Superior mesenteric artery. Citation: [13-19]

Cases	Age		Sex	Symptoms		Aortomesenteric angle	Management		Follow up
Case 1	17 years		Male	Upper abdominal pain, vomiting		7 degrees	Open Duodenojejunostomy		Pain resolved, occasional vomiting at one year
Case 2	17 years		Male	Postprandial bilious vomiting		12 degrees	Laparoscopic Duodenojejunostomy		Continues to have vomiting once in a month at one year
Case 3	31 years		Male	Postprandial upper abdominal pain		10 degrees	Open Duodenojejunostomy		Asymptomatic at one year
Case 4	20 years		Female	Postprandial non-bilious vomiting		11 degrees	Open Duodenojejunostomy		Asymptomatic at one year
Case 5	22 years		Male	Postprandial bloating, upper abdominal pain		10 degrees	Laparoscopic Duodenojejunostomy		Asymptomatic at one year
Case 6	17 years		Female	Upper abdominal pain, vomiting		12.5 degrees	Laparoscopic Duodenojejunostomy		Asymptomatic at one year
Case 7	45 years		Female	Upper abdominal pain		16.9 degrees	Open exploration laparotomy Strong’s procedure		Asymptomatic at four years
Case 8	44 years		Female	Abdominal pain, weight loss, nausea, vomiting		14.7 degrees	Open exploration laparotomy Strong’s procedure		Asymptomatic at two years
Case 9	73 years		Male	Nausea, bilious vomiting, colicky abdominal pain		23 degrees	Conservative		Asymptomatic at three months
Case10	70 years		Male	Nausea, upper abdominal pain vomiting, abdominal distention		11 degrees	Conservative, open gastrojejunostomy side-to-side Duodenojejunostomy		Asymptomatic at three months
Case 11	15 years		Male	Acute bilious vomiting, epigastric pain		14 degrees	Conservative		Asymptomatic at one month
Case 12	16 years		Male	Abdominal pain, nausea, on-bilious vomiting, foul-smelling vomiting, abdominal distention		20 degrees	Laparoscopic Duodenojejunostomy		Asymptomatic at eight days
Case 13	21 years		Female	Vague pain discomfort, vomiting, weight loss		20 degrees	Conservative		Asymptomatic at three months

The mean age of patients was 32 years. The male-to-female ratio was 7:6. Postprandial vomiting associated with upper abdominal discomfort was the most frequent occurrence. Significant weight loss was observed in two patients, and four patients had nausea during the presentation. SMAS confirmed proximal duodenal dilation associated with a narrowed aortomesenteric angle. Four patients with a diagnosis of SMA syndrome were treated conservatively, and the other nine were treated surgically with several types of surgery according to the evaluation of their condition. The duration of symptoms includes any management and follow-up duration to relieve the symptoms.

## Conclusions

This case describes a young woman with symptoms of intestinal obstruction diagnosed with SMAS. She was treated conservatively with TPN for one week and IV fluids until her bowel opened and the condition resolved. SMAS is an infrequent cause of high intestinal bowel obstruction and has many causes that reduce the angulation between SMA and AA. This case highlights that physicians must maintain a high index of suspicion to correctly diagnose this condition, which shares symptoms with many other conditions.
